# Do disability grants influence adherence to antiretroviral therapy?

**DOI:** 10.4102/ajod.v3i1.100

**Published:** 2014-04-23

**Authors:** Ashraf Kagee

**Affiliations:** 1 Department of Psychology, Stellenbosch University, South Africa

## Abstract

Anecdotal data suggest that some South Africans living with HIV who receive disability grants from the state deliberately default on their antiretroviral medication in an attempt to lower their CD4 count to remain eligible for grants. No actual empirical data however exist to show that disability grants act as such perverse incentives and are a valid reason for non-adherence. This article examines some of the complexities of antiretroviral adherence in the context of a resource-constrained environment. The multitude of structural barriers, including sometimes difficult patient-doctor conversations about the renewal of disability grants, shape patients’ experiences of the clinic environment and influence their adherence to care.

In 2009, a major national South African newspaper published a report stating that ‘driven by sheer poverty, scores of desperate AIDS victims are refusing life-saving treatment to get social grants’ (Govender 2009). The report also cited organisations such as the AIDS Consortium and the National Association of People Living with HIV and AIDS which stated that antiretroviral therapy users refused to adhere to their treatment regimens as they believed they would qualify for a disability grant if their CD4 count was below a specific level.

Antiretroviral therapy (ART) plays a crucial role in the maintenance of health of persons living with HIV. The data are unequivocal that the introduction of ART has saved millions of lives when patients maintain high levels of adherence to their medications (Parienti *et al*. 2008, Kimmel *et al*. 2013; Nsubuga, Maher & Todd 2013). The possibility that the desire to retain a disability grant may stand in the way of optimal adherence, constituting a perverse incentive to health maintenance, is therefore cause for concern.

Nattrass (2007) argued that South Africa’s disability policy might incentivise current recipients of disability grants to remain ill by deliberately not taking their medication, resulting in an increase in viral load and a reduction in CD4 count, thus making them eligible for the continuation of their grant. Nattrass however does not regard deliberate non-adherence as a significant problem. On the other hand, a study by Leclerc-Madlala (2006) appears to suggest that some may even deliberately become infected with HIV so as to receive a grant. A crucial question is whether, beyond anecdotal data, there exists evidence that disability grants serve as a perverse incentive for non-adherence to ART.

South Africa’s social welfare system awards grants to aged and disabled persons, and children. Adults from the age of 18 who are unable to work because of a mental or physical disability are eligible for a means-tested disability grant, consisting of a monthly payment from the state. The disability section of the *Social Assistance Amendment Act*, 2010 states that:

a person… (is) eligible for a disability grant, if he or she has attained the prescribed age and is, owing to a physical or mental disability, unfit to obtain by virtue of any service, employment or profession the means needed to enable him or her to provide for his or her maintenance. (South African Government 2010)

The grant is either permanent, requiring renewal every 5 years, or temporary, requiring renewal every year. In cases of persons living with HIV, it is a requirement that a medical officer makes a recommendation about the patient’s eligibility for a grant, taking into account CD4 count and functional status. Decisions about awarding disability grants to persons living with HIV are not uniform as some variability exists in terms of eligibility criteria (Swartz, Schneider & Rohleder 2006). Indeed, Venkataramani *et al*. (2010) showed that amongst South African disability grant recipients, grants did not work as intended as some persons who should have been ineligible to receive them continued to do so. Possible reasons for such continued receipt were poor oversight of grant administration or the collusion of medical staff to endorse grant eligibility when this was not warranted.

In the context of South Africa’s high unemployment rate, estimated at 25.6% in 2013 but which is widely considered to be substantially higher, disability grants contribute significantly to the financial well-being of the country’s households (Nattrass 2005). As there is no welfare provision for the unemployed, the disability grant is the only available grant for adults of working age.

In a mixed method study consisting of semi-structured interviews with persons living with HIV and public health doctors, focus group discussions with program managers and community workers, and a quantitative survey of ART users, De Paoli, Grønningsæter and Mills (2012) found that patients who were no longer eligible for grants had a substantially diminished household income. Some grants have come to be seen as highly desirable as a means of generating an income. Anecdotal accounts abound of the role HIV infection has played in providing persons living in poverty with a source of income via disability grants. Steinberg *et al*. (2002) reported for example on the results of a study of households affected by HIV, in which one interviewee stated:

I love this HIV, now at least with the grants I am trying… Yes I like this HIV/AIDS because we have grants to support us. The R110 child support grant and the R570 disability grant. I applied for another grant for R390 but they were asking too many questions. (n.p.)

Of the 216 ART-users in the study by De Paoli *et al*. (2012) 10% agreed with the statement that ceasing to take medications was a common strategy to becoming ill so that they could renew their grant. In qualitative interviews amongst a smaller sample, some participants reported subtle ways of influencing biological markers to enhance the likelihood of grant renewal. These methods included consuming alcohol or skipping doses prior to a clinic visit. Reduced financial circumstances associated with grant loss added to psychological stress but also made a healthy diet, necessary to support ART, inaccessible (De Paoli *et al*. 2012). To this extent, optimal adherence was characterised by circumstantial difficulties which, whilst not directly influencing adherence, created sub-optimal conditions for high levels of pill-taking.

In a longitudinal study, Venkaramani *et al*. (2010) investigated the extent to which disability grants constitute a barrier to adherence amongst a township sample outside Cape Town. Amongst both grant recipients and non-recipients, reported adherence was either perfect or close to perfect and no member of the sample indicated that they would stop ART to remain eligible for a grant. These authors concluded that ‘despite a high probability of grant loss… no individual reported imperfect adherence or an inclination to modify treatment for grants’ (Venkaramani *et al*. 2010:1396). However, a secondary finding of this study was that some ART users continued to receive grants despite being ineligible for them. This observation suggested that medical staff, whose task it was to determine eligibility for renewal of grants, may have out of sympathy for poor and unemployed patients, authorised renewal when this was not warranted from a strict legalistic point of view. Relatedly, the practice of medical officers not abiding by bureaucratic rules in the service of their patients was documented in clinical decision-making regarding eligibility for treatment (Bayer & Oppenheimer 2007). They called attention to instances when clinicians overrode administrative guidelines of rationing medications under conditions of scarcity and dispensed medication nonetheless (Bayer & Oppenheimer 2007).

In a study on structural barriers to ART adherence, HIV medical personnel indicated that the issue of disability grants was an important consideration in the dynamics of HIV care (Coetzee, Kagee & Vermeulen 2011). They reported that patients were often unaware that their grants were temporary rather than permanent, and that renewal was often not warranted because of improvements in patients’ health, ostensibly due to the effects of treatment. In cases where the health professionals believed that renewal was not warranted due to the patient’s improved health status after the expiry of the initial grant term, medical staff reported that conversations between themselves and patients about renewal of the grant sometimes led to ruptures in these relationships. In the context of several other barriers to adherence, such as long waiting times at clinics, overcrowding, linguistic barriers during consulting, and transport disruptions, such ruptures were seen by clinicians as potentially contributing to sub-optimal adherence to care (Coetzee *et al*. 2011). Barriers such as these created a difficult clinic context for the provision of care and cumulatively created and sustained structural barriers to adherence. Furthermore, loss of a grant appeared to have an indirect effect on health. In sum, despite existing anecdotal data on disability grants serving as perverse incentives for patients to be non-adherent so that they could evidence a low CD4 count, no firm evidence exists to support this assertion.

## Child-bearing to access disability grants: An analogous situation

An analogy to the supposed perverse incentive created by disability grants amongst ART users is the Child Support Grant (CSG) amongst poor young women. An argument has been advanced that since its introduction in South Africa in 1998, the CSG created an incentive for poor women to bear children so that they may gain access to an income which, in the context of high unemployment, is inaccessible to them. A study conducted by the Human Sciences Research Council in South Africa, refuted the hypothesis that CSG’s constituted a perverse incentive to teenage pregnancy (Makiwane *et al*. 2006). These authors found that the rate of teenage pregnancy started to decline prior to the introduction of the CSG, that increased rates of teenage pregnancy occurred across all social sectors, including those who did not qualify for the CSG on the means test, and that only a small proportion of teenage mothers received the CSG anyway. The authors concluded that there was no evidence that South African teenagers deliberately fall pregnant to access the CSG and to this extent no perverse incentive may be said to be in effect. The case of CSG’s provides a useful analogy in understanding non-adherence amongst ART users. It is probably unlikely, except in a few isolated cases, that persons living with HIV deliberately forgo their medication so that their viral load increases and CD4 count decreases in order to access disability grants. The cost of poor health and the risk of death associated with suboptimal adherence most likely eclipses the usefulness of such a strategy.

## Creating health-enabling conditions in a resource-constrained environment

By all accounts the creation of a health enabling environment is necessary to support sustained health behaviour amongst community members. Campbell and MacPhail (2002) define a health enabling community as ‘a social and community context that enables or supports the renegotiation of social identities and the development of empowerment and critical consciousness’ (Campbell & MacPhail 2002:334). In other words, a health-enabling community creates conditions that enable and support health-enhancing behaviour. The notion of perceived citizen power, a corollary of a health-enabling community (Campbell, Wood & Kelly 1999), is when citizens experience their needs, opinions and views as being respected and valued, and when they believe they have appropriate opportunities to make decisions in their social contexts (Campbell & MacPhail 2002).

The creation of health-enabling environments may hold the potential to enhance ART adherence. In a resource-constrained environment, simple cash transfers to patients to promote adherence have not been sufficiently researched to warrant recommendation, and their contribution to fostering a health-enabling environment is therefore unclear. However, direct funding targeted at specific problematic areas in patients’ lives may contribute to creating a health-enabling environment within which increased adherence is likely. Mukherjee *et al*. (2006), for example, showed that the combination of several structural and support strategies could enhance the likelihood of successful levels of adherence amongst Haitian ART users. These strategies included providing free medications and medical services to patients, integrating HIV services with primary care services, providing transport and food assistance to patients most in need as a way to overcoming these barriers, and providing psychosocial support to patients in the form of community health workers (Mukherjee *et al*. 2006).

In South Africa’s public health system ART services are provided at no charge to users. The free availability of ART followed several years of political activism against pharmaceutical companies to reduce the cost of essential life-saving medications and later against a government policy of AIDS-denialism (Geffen 2010).

In explaining adherence success in sub-Saharan countries, specifically Nigeria, Tanzania, and Uganda, Ware and colleagues argued that social capital played an important role in adherence behaviour (Ware *et al*. 2009). These authors call attention to the important role that social relationships play in preserving health and ensuring survival in resource-constrained environments, noting that ‘when health care is a scarce resource, illness imposes an extra burden on social intimates who must then assume responsibility for care’ (Ware *et al*. 2009:43). Caregivers thus have to invest time, effort, energy, as well as scarce financial resources to promote patients’ health. Such investment is likely to be more forthcoming if recovery is expected, rather than the continued physical decline and the eventual death of the patient. To this extent, in order for patients to maximise the social capital available to them, characterised as trust, cooperation, reciprocity, and sociability, good adherence is essential. Adherence, health improvement and maintenance permit patients to rely less on others, thus preserving social capital. Conversely, poor adherence and a lack of personal responsibility for health-promoting behaviour may erode social capital. ART adherence is thus a health priority and therefore a social priority, which led participants in Ware *et al.’*s (2009) sample to resort to finding funds for transport, food, and other necessities by a variety of means, including borrowing and begging, and sacrificing other important needs in the service of health maintenance. Taking personal responsibility for health in the context of high social capital therefore increases the likelihood that help will be available when the need arises in the future (Ware *et al*. 2009). In this argument the incentive of social capital and the motivation to preserve it in the event of future need stands in opposition to the presumed disincentive that a disability grant may represent when patients become well. Where tension exists between the need for retention of a disability grant in the context of poverty on one hand, and the threat of the erosion of social capital on the other, it appears to be more likely that the latter will hold sway.

## Disability grants and human rights

Social welfare for those unable to work is imperative in a humane society premised on individual, political, and socio-economic rights. The question of social grants for persons living with a chronic illness takes on an additional dimension in the context of a resource-constrained environment in which the possibility of employment is close to zero for those lacking in skills, training, and experience. In one possible scenario, qualifying ART-users would be eligible for a poverty-relief grant that would augment the disability grant, and even replace it when the latter is no longer renewable. Recipients would thus not be reliant solely on a disability grant, as it is necessarily time-limited and available only until the patient is deemed able to work according to the national guidelines. A poverty-relief grant may contribute to household income, offsetting the dire effects of poverty to a significant extent.

However, the provision of a poverty-relief grant brings into focus another problem, one which potentially contributes to social injustice: providing a financial payment to persons living with HIV may be seen as unfair to those not living with the virus. An appropriate question might be why persons living with HIV are more deserving of poverty-relief than their HIV-negative counterparts. With both groups equally impoverished, there is perhaps no compelling argument in favour of paying a cash amount to one group and not the other solely for the purpose of poverty-relief. Moreover, another unintended consequence may surface of HIV negative persons deliberately becoming infected to become eligible for a poverty-relief grant. Whilst no empirical data exist to suggest that deliberate infection is a widespread practice, some anecdotal data seem to indicate that it may be a concern in certain instances. For example, Nattrass (2004) cites examples of Zambian sex workers who charged $2.00 for sex with a condom and $4.00 without, increasing their risk of infection. They were reported as saying that they would rather die of AIDS than hunger (*Mail & Guardian*, 01–07 November 2002 cited in Natrass 2004:n.p.).

Another proposal to enable poor households to meet their basic needs, stimulate economic development, and promote family and community stability is the Basic Income Grant (BIG). The BIG Coalition is premised on a clause contained in the South African constitution, stating ‘[*e*]veryone has the right to have access to … social security, including, if they are unable to support themselves and their dependents, appropriate social assistance’ (Constitution of the Republic of South Africa, ch. 1, art. 27, ss. 26–28). Despite the constitutional provision for social security, nearly half of South Africans live in poverty (Armstrong, Lekezwa & Siebritz 2009), with most having no access to social security. Since the early 2000s the BIG Coalition has called for a universal income support grant which would provide citizens with a minimum level of income, enable poor households to meet their basic needs, and stimulate economic development (BIG Coalition 2009). As a discussion of the merits and demerits of BIG is beyond the scope of this article suffice it to say that this option appears to have faded from public discussion in recent years, and may not at this time be a credible solution to addressing the problems of adherence.

## Poverty alleviation

Both poverty relief grants and a basic income grant bring into focus an overarching feature of countries in sub-Saharan Africa, namely economic underdevelopment. In many countries in the global North, disabled persons have access to a range of services and resources, including subsidised housing, a guaranteed regular income, and psychosocial support (Committee on the Rehabilitation and Integration of People with Disabilities 2003). The same is not the case in countries in the global South, especially in sub-Saharan Africa where grants, if available, are much more limited in value. With an average GDP per capita of less than $6000.00, many sub-Saharan African countries are unable to provide social assistance of any meaningful value. South Africa with an average per capita GDP of $3745.00 (World Bank 2010) is considered the largest welfare state in the developing world (Bernstein 2005). The relationship between disease burden and economic development is reciprocal. Poverty increases the likelihood of a high disease burden in a society, and disease in turn exacerbates poverty. The relationship that better health contributes to intellectual and physical development and thus greater workplace development has been well-documented. As stated in a 2005 UN Report ‘AIDS deepens poverty and increases the number of poor at risk of infection, because those with the fewest resources have the least access to health-care services or health-related information’ (United Nations 2005:13). Conversely, living under impoverished conditions exacerbates the spread of HIV through sexual networks characterised by transactional sex, gender inequality, migrant labour, political conflict, and forced migration. For the reciprocal relationship between poverty and the spread of HIV to be disrupted, structural changes, including economic solutions are necessary. Whilst social grants may offer important and necessary short-term solutions for individuals and families struggling for survival, they are not likely to constitute permanent solutions to endemic poverty. The emphasis instead, has to be placed on education and training, job creation, and reducing stigma towards persons living with HIV. In the Diagnostic Overview of South Africa’s National Planning Commission (South African Government 2012), several challenges to economic development were identified. These were: too few South Africans in either formal or informal employment; sub-standard school education, especially for black South Africans; inadequate infrastructure which undermines developmental efforts; spatial challenges due to apartheid social planning that marginalise the poor; a resource-intensive growth path which is not sustainable; a dysfunctional public health system alongside a considerable disease burden; an under-performing public service; corruption in government; and social, class and racial divisions that characterise South African society (South African Government 2012). These social and economic problems are clearly interrelated and no single approach will result in their resolution. The development of solutions to these problems requires a complex and multifaceted approach utilising the expertise and skills of a variety of players, including the political elite, trade unions, researchers, civil servants, the business community, the non-governmental sector, and others. With the ultimate objective of increasing sustainable employment rates, it may be that persons living with HIV may find that employment opportunities do indeed exist for them, obviating the need for disability grants, except amongst those whose health is compromised.

## Implications for future research

There has been considerable descriptive research on adherence to ART in resource-constrained areas, although social, behavioural and policy interventions have remained untested in yielding optimal results in terms of high levels of clinic attendance and pill-taking. It is likely that careful case study research will yield useful data on the moment-to-moment decisions taken by ART users about the ways they balance the contextual demands of a resource-constrained environment with health-promoting imperatives such as adherence. In the absence of florid symptoms and in the context of competing demands on time and resources, medication adherence is seldom likely to be prioritised. Interventions related to creating health-enabling environments also warrant investigation, although it is doubtful that HIV-specific interventions alone are most appropriate given the high prevalence of other chronic illnesses in many poor communities. Interventions to enhance treatment adherence more broadly amongst persons living with chronic illnesses is an area in need of further research. Relatedly, the interface between individual decision-making and contextual and structural factors is also in need of theoretical development.

The policy implications for health promotion amongst persons living under conditions of poverty are complex. On the one hand, it has been shown that ART users in developing countries are as much or more adherent to their medication regimens than their counterparts in industrialised countries (e.g. Mills *et al*. 2006). Yet, there is no doubt that poverty creates conditions that affect health adversely. Poverty alleviation programmes may be the first port of call, but whether such programmes will necessarily result in health promotion is unclear. Social policies that create health enabling environments by addressing the structural conditions in communities in all likelihood stand the best chances of success, for example, school and workplace health promotion, screening, and intervention programmes, community health centres that engage in outreach activities, and infrastructural development such as housing and sanitation so that the rate of infectious diseases may be reduced. These are long-term interventions that will presumably have long term implications for health and health care, and eventually medication adherence.

## Concluding remarks

There is no direct empirical evidence for the notion that disability grants serve as a perverse incentive for patients to become non-adherent to their ART medication and thus compromise their health. When considering health decision-making, including adherent behaviour amongst patients in resource-constrained contexts such as South Africa, subtle ways in which the social and economic environment influences behaviour should however be noted. Clinic conditions may often impede the creation of health enabling environments, because of difficult structural and personal dynamics, including a lack of agreement between medical staff and patients about the appropriateness of grant eligibility and renewal. Such a mismatch of understanding should be seen together with other structural factors such as overcrowded clinics, staff burnout, food insecurity, stigma, lost wages, and varying levels of social support. By itself the disability grant mechanism is most likely not a perverse incentive to ART adherence, but the terrain in which many patients in sub-Saharan Africa find themselves, characterised by scarce resources, contributes to the multitude of challenges which ultimately influence health behaviours, including adherence.
